# Dedifferentiation and Redifferentiation of Follicular-Cell-Derived Thyroid Carcinoma: Mechanisms and Therapeutic Implications

**DOI:** 10.3390/biomedicines13122982

**Published:** 2025-12-04

**Authors:** You He, Zimei Tang, Ming Xu, Tao Huang

**Affiliations:** Department of Breast and Thyroid Surgery, Union Hospital, Tongji Medical College, Huazhong University of Science and Technology, Wuhan 430022, China; heyouwh@163.com (Y.H.); tangzimeiwh@163.com (Z.T.); mingxuwhuh@outlook.com (M.X.)

**Keywords:** thyroid carcinoma, genetic study, radioindine therapy, dedifferentiation, redifferentiation, biomarker

## Abstract

Follicular-cell-derived thyroid carcinoma, while typically associated with a favorable prognosis, can undergo dedifferentiation into poorly differentiated (PDTC) or anaplastic thyroid carcinoma (ATC), leading to enhanced aggressiveness and radioiodine resistance. This review systematically examines the genetic and molecular mechanisms driving this pathological progression, highlighting the roles of key mutations—such as BRAF, RAS, TERT, and TP53—and the disregulation of signaling pathways, including MAPK and PI3K/AKT. These alterations promote the loss of thyroid-specific functions, including iodide metabolism, and correlate with poor clinical outcomes. In recent years, therapeutic strategies aimed at tumor redifferentiation have emerged as a promising approach for radioiodine-refractory disease. We summarize recent advances in the use of targeted agents, particularly BRAF and MEK inhibitors, to restore radioiodine avidity and improve treatment response. While early clinical studies show encouraging results, including tumor shrinkage and restored RAI uptake in selected patients, challenges such as treatment resistance and patient selection remain. Future efforts should focus on refining molecular stratification, developing rational combination therapies, and integrating novel modalities such as immunotherapy to overcome resistance. A deeper understanding of redifferentiation mechanisms not only provides insights into thyroid cancer progression but also supports the development of personalized treatment strategies for high-risk patients.

## 1. Introduction

Thyroid carcinoma, the most prevalent endocrine malignancy, is predominantly derived from thyroid follicular cells and includes papillary, follicular, and Hurthle cell carcinoma [1]. Differentiated thyroid carcinoma (DTC) accounts for approximately 90% of thyroid cancers, with a five-year survival rate exceeding 98% in early-stage cases [2]. However, dedifferentiation into poorly differentiated (PDTC) or anaplastic thyroid carcinoma (ATC) drastically worsens prognosis, with five-year survival rates of 76% and 7%, respectively.

Our comprehension of the fundamental genetics and molecular biology of follicular-cell-derived thyroid carcinoma has undergone significant transformation over the last two decades, following James A Fagin’s initial proposition of a molecular model of thyroid cancer progression in 1992 [3]. Dedifferentiation involves progressive loss of thyroid-specific functions, such as iodine uptake, driven by genetic mutations and alterations in signaling pathways. Recent advancements in genomic technologies have identified mutations in genes like BRAF, RAS, TERT, and TP53, as well as dysregulated pathways (e.g., MAPK, PI3K/AKT), as critical contributors to this process [4]. These findings not only deepen our understanding of tumor progression but also highlight potential therapeutic targets.

**Figure 1:**
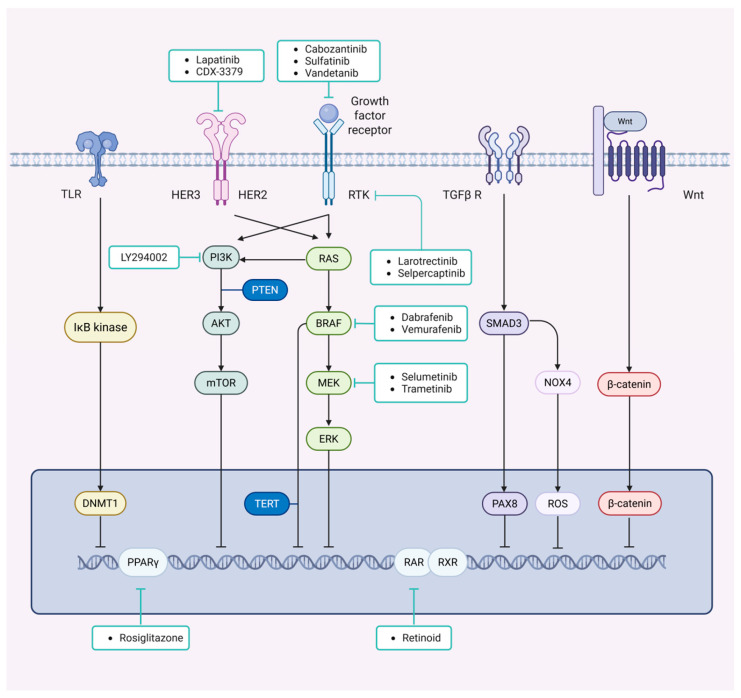
Schematic diagram of dedifferentiation mechanisms and redifferentiation therapy in thyroid cancer. TLR, Toll-like receptors; DNMT1, DNA (cytosine-5)-methyltransferase 1; HER, human epidermal growth factor receptor; PI3K, phosphoinositide 3-kinase; AKT, protein kinase B; mTOR, mammalian target of rapamycin; PTEN, phosphatase and tensin homolog; RAS, rat sarcoma; BRAF, B-Raf proto-oncogene, serine/threonine kinase; MEK, mitogen-activated extracellular signal-regulated kinase; ERK, extracellular regulated protein kinase; SMAD3, SMAD family member 3; PAX8, paired box 8; NOX4, NADPH oxidase 4; ROS, reactive oxygen species; Wnt, Wnt signaling pathway. Arrows indicate activation of pathway molecules, while lines without arrowheads denoted direct actions on DNA. Created in BioRender. You, H (2025). https://BioRender.com/vhhzyjy (accessed on 29 October 2025).

Emerging redifferentiation therapies aim to restore iodine uptake by targeting these molecular alterations, offering a promising strategy for radioiodine-refractory (RAI-R) thyroid cancer [5]. This therapy has shown promising results in limited clinical trials, exhibiting tumor reduction and disease stabilization in certain patients, which offers a novel treatment avenue for individuals with RAI-R thyroid cancer. This review discusses the molecular mechanisms of dedifferentiation and therapeutic strategies for redifferentiation, emphasizing their clinical implications in diagnosis and treatment (Figure 1).

**Literature Search**: We performed a comprehensive literature review utilizing PubMed and MEDLINE to gather both English and non-English publications from 1990 to 2025, which we subsequently analyzed. Our primary focus was on the mechanisms underlying dedifferentiation and redifferentiation in DTC, alongside the assessment of differentiation levels and the approaches to redifferentiation therapy. To ensure the inclusion of pertinent studies, we used the “related articles” feature on PubMed to uncover additional relevant literature. We placed particular importance on the most recent studies published in the last five years. The key search terms include thyroid carcinoma, cell dedifferentiation, biomarker, redifferentiation, and radioiodine therapy.

## 2. Dedifferentiation

### 2.1. Molecular Mechanisms of Dedifferentiation

The pathogenesis and progression of DTC involve multiple genetic alterations, including somatic mutations, gene rearrangements, gene amplifications, and copy number variations. These genetic events are typically mutually exclusive and occur within specific tumor subtypes. Key signaling pathways implicated include the MAPK pathway, the PI3K/AKT pathway, the WNT pathway, the TGF-β/SMAD pathway, and the NF-κB pathway.

#### 2.1.1. Genetic Mutations

BRAF and RAS gene mutations are core drivers of thyroid cancer; they are generally mutually exclusive and induce varying degrees of MAPK pathway activation. Papillary thyroid carcinoma (PTC) is predominantly associated with BRAF mutations, whereas follicular thyroid carcinoma (FTC) is mainly linked to RAS mutations. BRAF^V600E^ mutations, found in approximately 59% of DTCs, lead to constitutive activation of the MAPK pathway, promoting aggressive tumor behavior and reducing iodine uptake. Conversely, RAS mutations, found in about 13% of DTCs, activate both the MAPK and PI3K/AKT pathways, contributing to a less aggressive phenotype [6]. In DTC, increased MAPK pathway flux negatively correlates with thyroid differentiation status [7]. Consequently, PTCs typically demonstrate higher MAPK pathway activity and diminished differentiation compared to FTCs, which may lead to divergent clinical outcomes in response to targeted therapies and radioactive iodine treatment.

Approximately 16% of DTCs are driven by gene rearrangements, primarily involving receptor tyrosine kinases (RTKs), such as RET, NTRK3, NTRK1, and ALK [6]. These fusion oncoproteins interact with RTK kinase domains, leading to prolonged downstream signaling. RET gene rearrangements are identified in approximately 7% of PTC cases, predominantly involving CCDC6-RET (RET/PTC1) and NCOA4-RET (RET/PTC3) gene fusions [8]. TCGA studies indicate that RET fusions are mutually exclusive with point mutations in BRAF or RAS, as well as BRAF fusions [9]. RTK gene fusions are significantly more prevalent in pediatric PTC (60–70%) [10] and radiation-induced PTC (40–70%) [11] compared to adult PTC (~15%). RET fusions are the most common genetic alterations in diffuse sclerosing papillary thyroid carcinoma (DSPTC) and are independent risk factors associated with aggressive histopathological features and higher recurrence rates [12]. The PAX8/PPARγ gene rearrangement is observed in 27% of FTC, generally exhibiting mutual exclusivity with RAS mutations [13].

The TERT promoter-driven telomerase reverse transcriptase maintains the protective telomeric sequences at chromosomal termini; mutations within this region can result in cellular immortalization and enhance oncogenic invasiveness. The prevalence of TERT mutations in DTC is approximately 9%, with higher frequencies observed in PDTC and ATC, at 61% and 65%, respectively, and they frequently co-occur with BRAF or RAS mutations [6]. Additionally, TERT promoter mutations are observed as subclonal variants in DTC, whereas in PDTC and ATC, they are clonal, indicating a highly immortalized oncogenic progression pathway [14]. Similarly, TP53 mutations, present in 65% of ATCs, disrupt tumor-suppressive functions, including apoptosis and cell cycle regulation, facilitating dedifferentiation [4]. TERT (83%) and TP53 (71%) mutations represent the most prevalent alterations in established (immortalized) thyroid cancer cell lines, detected in nearly all early dedifferentiated PTC cases [5].

In addition, there are numerous low-frequency oncogenic mutations that collectively shape the comprehensive mutational landscape of DTC. EIF1AX, identified as a novel oncogenic driver, exhibits a higher mutation prevalence in PDTC and generally demonstrates mutual exclusivity with MAPK pathway mutations [14]. Although mutations in DNA repair genes such as CHEK2 and PPM1D occur at low frequencies, they frequently co-occur with MAPK pathway alterations, potentially facilitating the progression from DTC to more invasive phenotypes [6]. In advanced tumors, mutually exclusive mutations are prevalent among components of the SWI/SNF chromatin remodeling complex (e.g., ARID1A, ARID2) and epigenetic regulators (e.g., KMT2C, CREBBP), leading to downregulation of thyroid transcription factors (NKX-2, FoxE1, Pax8), promoting tumor dedifferentiation and resistance to radioactive iodine (RAI) therapy [15]. Moreover, next-generation sequencing has identified novel gene rearrangements involving BRAF, NTRK, and ALK, alongside copy number variations affecting cell cycle regulators such as CDKN2A/B [4].

**Figure 2:**
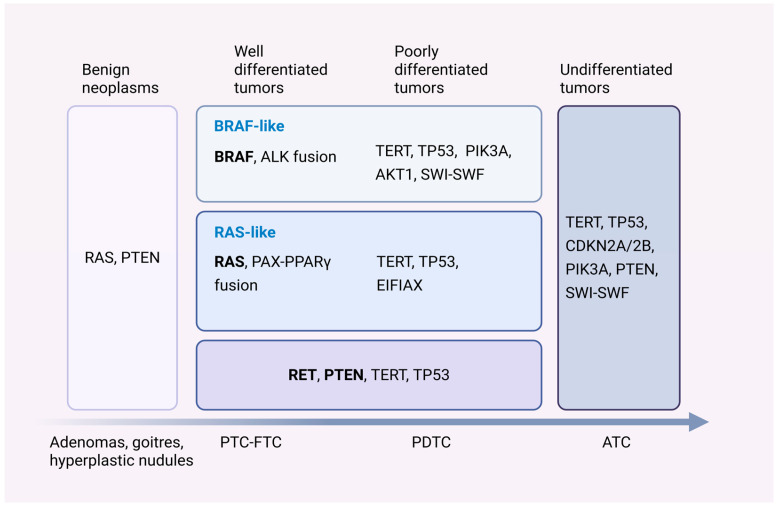
Genetic Alterations in the Progression of Thyroid Carcinoma. Genetic events associated with tumor initiation are indicated in bold. BRAF and RAS mutations are common early events. While the BRAF^V600E^ mutation is diagnostically significant (detected only in malignant tumors), RAS mutations have also been identified in benign specimens. Some thyroid cancers are driven by RET fusions or PTEN loss, with the latter also observed in benign samples. Among genetic events linked to tumor progression, mutations such as those in TP53 show little association with initiation factors, whereas others are highly correlated with specific initiating events. Examples include RAS with EIF1AX, BRAF with PIK3CA, BRAF with AKT1, and BRAF with SWI/SNF complex genes. Mutations in TP53, loss of p16 (CDKN2A), or alterations in SWI/SNF complex genes may drive rapid tumor progression to anaplastic thyroid carcinoma (ATC). Arrows at the bottom of the figure illustrate a trend over time. Created in BioRender. You, H (2025). https://BioRender.com/vhhzyjy (accessed on 29 October 2025).

In the progression of DTC, subsequent accumulating mutations exhibit distinct patterns of mutual exclusivity or co-occurrence with initial BRAF/RAS mutations. PTCs harboring BRAF^V600E^ mutations often display concurrent mutations in PIK3CA and AKT1; mutations in the SWI/SNF complex have also been identified in in vivo models coexisting with BRAF^V600E^ mutations, jointly promoting tumor progression [15]. RAS mutations are frequently associated with loss of function of PTEN and EIF1AX mutation [16] (Figure 2).

#### 2.1.2. Main Pathways

The MAPK signaling pathway serves as a principal pathway in the development and progression of thyroid carcinoma, primarily activated through mutations in the BRAF and RAS genes. This pathway includes RTK, RAS, RAF, MEK, and ERK components [14]. The BRAF^V600E^ mutation signals as a monomer that is unresponsive to ERK-mediated negative feedback, which leads to sustained MAPK pathway activation. In contrast, RAS mutation activates both MAPK and PI3K/AKT pathways. They signal through RAF dimers, which are subject to ERK negative feedback, resulting in attenuated pathway output. In genetically modified murine models of ATC, specific mutation combinations are observed, such as BRAF and PIK3CA [17], BRAF and TP53 [18], RAS and NF2 [19], RAS and TP53 [20], as well as PTEN and TP53 [21], which closely mimic human tumor profiles. Targeting this pathway with BRAF or MEK inhibitors has shown potential in reversing dedifferentiation and restoring iodine uptake in certain refractory cases.

The PI3K/AKT pathway, comprising components such as PIK3CA, AKT1, PTEN, and mTOR signaling complexes, plays a complementary role in tumor progression. This pathway is typically activated through binding with RAS and the catalytic subunit p110 (primarily PIK3CA and PIK3CB), leading to the generation of phosphatidylinositol-3,4,5-trisphosphate (PIP3). This lipid mediator recruits AKT to the plasma membrane, where it initiates downstream activation of mTOR and other related proteins. PTEN acts as a crucial negative regulator of this pathway. Mutations in PIK3CA or loss of PTEN lead to AKT and mTOR activation, driving cell proliferation. These alterations are frequently observed in PDTC and ATC, often co-occurring with MAPK pathway mutations. Specifically, PI3KA and AKT mutations are mainly linked with BRAF^V600E^ mutations, while PTEN loss is strongly linked to RAS and NF1 mutations [14].

The WNT signaling pathway encompasses proteins encoded by the CTNNB1 (β-catenin), AXIN1, and APC genes, which are critical mediators in cell adhesion and transcriptional regulation [14]. APC interacts with β-catenin and recruits kinases such as casein kinase I and glycogen synthase kinase-3 (GSK3). WNT signaling inhibits the degradation of CTNNB1, allowing its translocation to the nucleus, where it functions as a transcriptional co-activator, modulating NIS localization and suppressing iodine uptake in thyroid carcinoma cells. TERT acts as a positive regulator within this pathway, leading to its activation and the promotion of anti-proliferative signals.

TGF-β/SMAD pathway involves transforming growth factor-beta (TGF-β) and its downstream signaling mediator SMAD, which play crucial roles in the regulation of cell proliferation, differentiation, and invasiveness. Its expression and activity are correlated with tumor invasion, lymph node metastasis, and BRAF mutation status. The BRAF^V600E^ mutation promotes activation of an autocrine TGF-β loop, leading to the suppression of the sodium/iodide symporter (NIS) [22].

NF-κB signaling pathway promotes tumor progression by regulating proliferation and anti-apoptotic mechanisms; simultaneously, several oncogenic proteins upregulated within this pathway can also be activated by mutations in the MAPK pathway, such as BRAF^V600E^, RAS, and RET fusion [23].

#### 2.1.3. Epigenetic Modifications

DNA methylation, histone modifications, and non-coding RNAs contribute to thyroid cancer development and progression. DNA methylation levels vary across subtypes: PTC shows the lowest frequency, FTC exhibits a high hyper-/hypomethylation ratio, potentially linked to differential BRAF/RAS mutation burdens [6,24], while ATC displays global hypomethylation with CpG island hypermethylation [25,26]. Histone modifications participate in early tumorigenesis. Levels of H3K18ac and H3K9ac/K14ac are higher in PTC and FTC than in ATC, suggesting their loss may enable progression [27]. Acetylation downregulates thyroid differentiation genes (e.g., SLC5A5, TG, TPO) via transcription factor regulation. Combined histone deacetylase (HDAC) and MAPK/PI3K pathway inhibition enhances antitumor effects in multiple thyroid cancer models [28]. Non-coding RNAs also regulate thyroid carcinogenesis, representing potential diagnostic and therapeutic targets. miRNAs (e.g., miR-146b, -221, -222) are upregulated in PTC and promote progression by targeting PTEN or modulating MAPK/PI3K signaling [29,30,31,32]. lncRNAs such as HOTAIR and NEAT1 drive proliferation and migration via Wnt signaling or miRNA interactions [33]

### 2.2. Clinical Diagnosis

The clinical diagnosis and risk stratification of DTC dedifferentiation relies on a multi-faceted approach that integrates patient demographics, comprehensive laboratory/imaging findings, and molecular profiling. 

#### 2.2.1. Clinical Characteristics

Several clinical factors help predict dedifferentiation in DTC. Age over 55 years is a significant independent risk factor for progression to aggressive phenotypes like ATC [34]. Male gender is also associated with more aggressive disease presentation [35], despite the overall higher incidence of thyroid cancer in females [36]. The occurrence of undifferentiated carcinoma occurs equally across genders, suggesting complex sex-specific influences [37].

Dedifferentiation entails significant behavioral changes: ATC shows accelerated growth, higher rates of lymph node metastasis and extraglandular invasion, and markedly reduced iodine uptake compared to DTC, with PDTC representing an intermediate state [38]. Even typically indolent papillary thyroid microcarcinomas (PTMC) may occasionally dedifferentiate, leading to metastatic disease [34].

#### 2.2.2. Diagnostic Criteria

The diagnosis of DTC relies on a multimodal approach. Serum biomarkers, including calcitonin, thyroglobulin (Tg), thyroglobulin antibody (TgAb), and thyroid-stimulating hormone (TSH), are valuable for postoperative monitoring but offer limited utility in preoperative diagnosis or risk stratification [39]

Imaging plays a central role in tumor assessment. Ultrasound remains the primary modality for evaluating nodules < 1 cm, achieving 64–77% sensitivity and 82–90% specificity based on features such as echogenicity, margins, and calcification patterns [40]. DTC demonstrates distinct sonographic features that correlate with differentiation status: well-differentiated tumors typically present as hypoechoic solid nodules, while poorly differentiated and anaplastic carcinomas show increasing heterogeneity, irregular borders, and larger dimensions [41]. CT imaging better characterizes invasive features, including local extension and nodal metastasis. For metastatic evaluation, nuclear medicine techniques provide functional assessment, with ^123^I/^131^I SPECT used for radioiodine-avid disease [42] and [^18^F]FDG PET/CT reserved for radioiodine-refractory cases [43].

Ultrasound-guided fine-needle aspiration biopsy (FNAB) represents the diagnostic gold standard, though indeterminate results occur in 5–20% of cases, particularly with follicular-patterned lesions [44]. This diagnostic gap underscores the growing importance of molecular biomarkers in refining preoperative diagnosis, staging, and therapeutic planning.

#### 2.2.3. Gene Sequencing

The 2015 American Thyroid Association guidelines recommend BRAF mutation or seven-gene panel testing (BRAF, RAS, RET/PTC, PAX8/PPARγ) to further inform surgical planning [38]. While BRAF^V600E^ correlates with aggressive features, its prognostic specificity is limited. Notably, co-occurrence of BRAF with TERT, PIK3CA, TP53, or AKT1 mutations significantly increases metastasis and recurrence risk [45,46]. The newly released 2025 ATA guidelines introduce a refined, risk-stratified molecular classification system, defining low-risk (RAS, BRAF ^K601E^, PAX8/PPARγ), intermediate-risk (BRAF ^V600E^, NTRK3 fusions, RET fusions), and high-risk (TERT promoter, TP53, AKT1, PIK3CA) alterations [47].

The mutually exclusive oncogenic drivers BRAF^V600E^ mutation and RAS mutations in DTC induce divergent signaling pathways. DTC can be stratified into BRAF^V600E^-like (BRL) and RAS-like (RL) molecular subtypes via BRAF-RAS signature (BRS) scoring. RL tumors typically harbor BRAF fusions, RET/ETV6-NTRK rearrangements, non-V600E BRAF mutations, or PAX8/PPARγ fusions, often presenting as follicular-variant PTC with preserved differentiation and favorable prognosis [6]. In contrast, BRL tumors (e.g., tall-cell variant) demonstrate aggressive behavior, frequent nodal metastasis, and reduced survival (82% vs. 98% in classic PTC) [48]. RAI-refractory tumors with RAS-like characteristics, such as RAS mutations [49,50] or BRAF^K601E^ [51], may respond better to redifferentiation therapies. A third transcriptional class, Non-BRAF/RAS-like (NBNR), defined by DICER1/EIF1AX/EZH1/IDH1/SPOP mutations or PAX8/PPARγ/THADA fusions, shows lower invasiveness [52].

The Thyroid Differentiation Score (TDS), quantifying 16 thyroid metabolic genes (e.g., TG, TSHR, TPO, SLC5A5), strongly correlates with BRS and RAI avidity. The significance of TDS lies in its ability to position tumors along a continuum of differentiation; tumors exhibiting high TDS levels are generally more responsive to RAI therapy [53]. RL-PTC exhibits higher TDS than BRL-PTC, while ATC shows global TDS suppression and lost TDS-BRS correlation, with mRNA levels of TG, TSHR, TPO, PAX8, SCL26A4, DIO1, and DUOX2 genes significantly suppressed [14]. Combining TDS and BRS may serve as a valuable tool for assessing the dedifferentiated state of DTC.

Despite molecular advances, current classifiers show limited prognostic precision. Future efforts should refine stratification through single-gene resolution and integrated biomarkers to address the dramatic survival disparities among PTC (98%), PDTC (76%), and ATC (7%) five-year survival.

## 3. Redifferentiation

### 3.1. Molecular Mechanisms of Redifferentiation

Following total thyroidectomy, high-risk DTC patients typically undergo radioactive iodine (RAI) therapy [54]. However, 5–15% develop RAI resistance (RAIR), rising to 50% in aggressive subtypes [55]. RAIR is defined by impaired iodine uptake or disease progression within one year post-RAI [56] and correlates with poor survival: 66% at 5 years [57] and 10% at 10 years for metastatic disease [58].

RAIR primarily results from tumor dedifferentiation, characterized by functional loss of the sodium/iodide symporter (NIS) [59]. Key pathways such as RTK/BRAF/MAPK and PI3K-AKT drive NIS suppression through genetic and epigenetic alterations [13,23]. Targeted inhibition of these pathways—using agents like dabrafenib, vemurafenib, or trametinib—has shown promise in restoring NIS expression and RAI avidity [60]. Compared to chronic tyrosine kinase inhibitor regimens, such redifferentiation strategies involve shorter courses (4–6 weeks), reduced toxicity, and lower resistance risk, positioning them as a viable therapeutic option for RAIR patients.

### 3.2. Therapeutic Regimen

#### 3.2.1. Targeting the MAPK Pathway

Multiple studies indicate that targeting the MAPK pathway, specifically MEK and BRAF, can restore radioiodine uptake in DTC patients. Early trials with the MEK inhibitor selumetinib demonstrated objective responses in some patients, particularly those with RAS mutations [49]. However, subsequent investigations were discontinued due to toxicity and insufficient improvement in iodine uptake [61,62]. BRAF inhibitors, such as dabrafenib and vemurafenib, have also shown therapeutic benefits [63,64]. Notably, some RAI-R patients experienced tumor regression with vemurafenib monotherapy, suggesting mechanisms beyond restored iodine uptake may contribute to tumor control.

Given the partial dependence of iodide transporter regulation on ERK signaling and feedback mechanisms limiting downstream effects, the efficacy of monotherapy is often constrained. Consequently, combination strategies have become a focus of research. The combination of dabrafenib and trametinib (DT) is currently the sole FDA-approved targeted regimen for advanced BRAF^V600E^-mutant thyroid cancer, significantly improving patient survival [65]. Nevertheless, challenges persist in achieving effective redifferentiation. Jaber et al. reported that effective restoration of RAI uptake was achieved in some RAS-mutant patients after MEK inhibition, but not in BRAF^V600E^-mutant patients treated with the dabrafenib and trametinib (DT) combination [66]. A Phase II multicenter study found no significant difference in objective response rates between dabrafenib monotherapy and DT combination therapy [67].

Furthermore, case reports suggest that the redifferentiation induced by combination therapy may be transient. A patient with BRAF^K601E^ mutation and distant metastases exhibited enhanced iodine uptake in metastatic lesions following combined DT and ^131^I therapy. However, uptake rapidly declined after treatment cessation, indicating a potentially temporary effect [51].

Beyond BRAF and MEK inhibitors, other targeted agents also show potential for redifferentiation. Selective NTRK inhibitor larotrectinib increased iodine uptake in pulmonary metastases in patients with EML4-NTRK3 fusions [68], while RET inhibitor selpercatinib showed efficacy in adult and pediatric PTC with RET fusions [69,70]. Future strategies involving the combination of multikinase inhibitors with MAPK pathway inhibitors or sequential treatment approaches may further enhance therapeutic responses. Completed and ongoing clinical trials related to redifferentiation therapy are summarized in Table 1 and Table 2.

#### 3.2.2. Beyond MAPK Pathway

Resistance to BRAF and MEK inhibitors presents a major therapeutic challenge, primarily mediated through RTK overexpression, paradoxical MAPK reactivation, and PI3K pathway hyperactivation [54]. To overcome these resistance mechanisms, combination strategies targeting HER3, PI3K, or immune checkpoints have shown considerable promise.

Preclinical models indicate that erbB-3 (HER3) receptor activation counteracts BRAF inhibitor effects on the MAPK pathway [75], while HER3 blockade with lapatinib restores MAPK pathway sensitivity. The anti-ER3 antibody CDX-3379 enhanced RAI uptake in a subset of BRAF^V600E^-mutant RAIR patients, with ARID2 mutations in the SWI/SNF complex identified as a potential resistance mechanism in non-responders [76].

PI3K inhibitors promote RAI uptake by upregulating the NIS via PAX8 activation. An ongoing Phase I trial (NCT04462471) is assessing BRAF plus PI3K inhibition in BRAF^V600E^-mutant RAIR patients. mTOR inhibitors similarly enhance differentiation through TTF-1 activation [77], and sorafenib-mTOR combination has shown efficacy in Phase II RAIR-DTC studies [78].

Immunotherapy combinations have also proven effective. Adding pembrolizumab to dabrafenib-trametinib (DT) significantly improved survival [79], while a PD-L1-high ATC patient with NRAS^Q61R^/BRAF^D594N^ mutations achieved complete remission after DT-sintilimab combination, enabling curative resection [80].

Nuclear receptor agonists provide additional redifferentiation avenues. PPAR-γ agonists improve RAI uptake in patients with high tumor PPAR-γ expression [81], while retinoids restore RAI avidity in 40–50% of RAIR cases, though their limited monotherapy efficacy warrants investigation in rational combinations [82].

### 3.3. Preclinical Research

Multiple combination strategies involving MAPK inhibitors are under preclinical investigation. Hui et al. demonstrated that the Pin inhibitor API-1 enhances BRAF inhibitor sensitivity in BRAF-mutant thyroid cancer by reducing HER3-mediated feedback activation of MAPK/ERK and PI3K/AKT pathways [83]. Using mESC-derived thyroid cancer organoids, Lasolle et al. showed that combined MAPK and PI3K inhibition reverses BRAF^V637E^-induced dedifferentiation in mouse cells and restores thyroid follicular structure and function in vitro [84]. Pita et al. further reported that triple therapy with CDK4/6 plus BRAF/MEK inhibitors achieves complete proliferation arrest in thyroid cancer cell lines while preventing resistance emergence [85].

Beyond the MAPK pathway, other signaling axes have also become focal points in redifferentiation research. In DTC, BRAF mutation-driven TGF-β secretion inhibits TGF-β/Smad signaling and downregulates NIS expression, with NOX4-derived ROS playing a critical role in this process. Elevated NOX4 levels, particularly in BRAF^V600E^ PTC, correlate with dedifferentiation and may predict RAI response [86]. The PKB inhibitor GDC-0941 also enhances iodine uptake in RET or BRAF^V600E^-mutant DTC models [87].

Epigenetic regulation represents another significant area of investigation. In vitro studies indicate that the combination of histone deacetylase inhibitors (HDACi) and MAPK inhibitors induces significant differentiation effects in BRAFV600E-mutated cells, with this effect being further enhanced by TSH [88]. The demethylating agent 5-azacytosine can restore NIS expression and iodine uptake function in cell lines with highly methylated NIS [89], while silencing miR-146b/miR-21 or selumetinib-mediated miRNA modulation reestablishes thyroid gene expression and NIS function [90]. Research has further confirmed that the MEK inhibitor selumetinib can restore NIS expression by downregulating specific miRNA levels [91]. Moreover, inhibiting GLI1 expression upregulates endogenous NIS, enhancing RAI uptake and ^131^I-mediated cytotoxicity, suggesting its potential as a novel redifferentiation strategy [92].

### 3.4. Therapeutic Assessment

After redifferentiation treatment, ^123^I scintigraphy and repeat ^124^I-PET are typically employed to assess iodine uptake. Patients exhibiting a regional target/background ratio exceeding 4 and an iodine uptake twice that of the average hepatic uptake on post-treatment ^123^I SPECT/CT are classified as responders and are eligible for ^131^I therapy. Imaging with ^[18F]^ FDG-PET/CT is conducted 3 to 12 months post-^131^I treatment, evaluated according to RECIST 1.1 [93] and PERCIST 1.0 criteria [94]. Serum levels of TSH, thyroglobulin, and thyroglobulin antibodies are monitored after 12 months to assess treatment efficacy [74].

Some researchers have identified elevated Tg levels post-targeted therapy as biomarkers for redifferentiation and subsequent RAI treatment response. However, serum Tg levels are influenced by both TSH and TgAb concentration. Elevated Tg may indicate disease progression, tumor redifferentiation, or tumor cell lysis, making it an imperfect differentiation marker in certain clinical contexts. Plasma drug monitoring aids in guiding clinical practice for redifferentiation therapy, with evidence linking dose-limiting toxicity (DLT) to trametinib and RAI reuptake to dabrafenib plasma exposure [95]. A direct correlation has been observed between the evaluated thyroid differentiation score (eTDS) and MAPK pathway inhibition, as well as RAI affinity in clinical specimens, suggesting that molecular characteristics may offer a more precise and comprehensive approach to quantifying thyroid differentiation and predicting RAI response [63]. Further studies with larger patient cohorts are necessary to validate this correlation and to establish a differentiation scale for thyroid cancer through genetic sequencing, enhancing clinical relevance.

## 4. Conclusions

This paper provides a comprehensive review of the mechanisms of dedifferentiation and redifferentiation in follicular-cell-derived thyroid carcinoma and their clinical significance. Studies have shown that the dedifferentiation of DTC is progressively achieved by mutations in several genes and alterations, and the most important genes include BRAF, RAS, RET, TERT, etc., which promote the progression of thyroid cancer cells from DTC to PDTC or even ATC through activation of the signaling pathways such as MAPK and PI3K/AKT, respectively. This process is usually accompanied by alterations in tumor biological behavior, manifested by increased aggressiveness, elevated risk of metastasis, and enhanced resistance to conventional treatments (e.g., radioactive iodine therapy), leading to a significant deterioration in prognosis. There is currently no consistent clinical definition of RAI-R, and existing criteria only predict the likelihood that a tumor is RAI-R. Standardized RAI uptake scans, as well as risk stratification of patients using genetic characteristics exhibited by aggressive or poorly differentiated tumors, are important.

With the deepening of molecular biology research, redifferentiation therapy targeting these mutated genes and pathways has become an emerging therapeutic strategy, especially for those thyroid cancer patients who are refractory to radioactive iodine therapy. Targeted agents such as BRAF and MEK inhibitors have shown positive therapeutic effects in some patients with RAI-R thyroid cancer by restoring the uptake capacity of radioactive iodine. This redifferentiation therapy strategy offers new hope for improving survival in these patients, and some patients have demonstrated tumor shrinkage and stable disease after treatment. However, although redifferentiation therapy has shown some efficacy in small-scale clinical studies, its long-term efficacy and drug resistance issues still need to be further validated by large-scale clinical trials.

Future research should focus on the following aspects: More personalized therapeutic strategies should be developed for patients with thyroid cancer of different molecular subtypes, including determining the mutation profiles of patients by gene sequencing to guide targeted drug administration. Second, therapeutic regimens combining multiple targeted drugs (e.g., BRAF and MEK inhibitors with PI3K/AKT inhibitors) should be the focus of future research to overcome the problem of resistance that may arise from single drugs. Combining novel therapeutic tools such as immunotherapy may further enhance the efficacy of redifferentiation therapy. A standardized treatment effect assessment system and improved biomarker detection methods will help to more accurately assess patients’ responses to treatment and optimize subsequent treatment regimens.

Overall, studies on the mechanisms of dedifferentiation and redifferentiation in follicular-cell-derived thyroid cancer have provided an important theoretical basis for understanding the progression of the disease and its treatment. With the continuous development of molecularly targeted drugs, redifferentiation therapy is expected to become an important treatment option for patients with RAI-refractory thyroid cancer and to further improve the prognosis of such high-risk patients.

## Figures and Tables

**Table 1:** Targeted redifferentiation approaches for radioiodine-refractory thyroid cancer (RAlR-TC) treatment.

Authors	Drug Targets	Therapy	Patients (*N*)	Oncogenic Driver (*N*)	Restored RAI Uptake (*N*)	RECIST Response	Partial Response [*N* (%)]
Ho et al. [49]	MEK	Selumetinib	20	BRAF-V600E (9)	4	At 6 months: 5 PR, 3 SD	5 (25)
				NRAS (5)	5		
				RET (3)	2		
				WT (3)	3		
Rothenberg et al. [64]	BRAF	Dabrafenib	10	BRAF-V600E	6	At 3 months: 2 PR, 4 SD	2 (20)
Huillard et al. [71]	BRAF	Vemurafeni, dabrafenib	1	BRAF-V600E	1	At 3 months: 1 PR	1 (100)
Jaber et al. [66]	MEK and/or BRAF	Selective dabrafenib, trametinib and/or vemurafenib; investigational MEKI	13	BRAF-V600E (9) NRAS (2) KRAS (1) WT (1)	8	At 8.3 months: 3 PR, 5 SD	3 (23)
Dunn et al. [63]	BRAF	Vemurafenib	10	BRAF-V600E	4	At 6 months: 2 PR, 2 SD	4 (25)
Iravani et al. [72]	MEK and/or BRAF	Dabrafenib +/− Trametinib; Vemurafenib + Cobimetinib	6	BRAF-V600E (3)	3	At 3 months: 3 PR, 1 SD	3 (50)
				NRAS (3)	1		
Leboulleux et al. [51]	MEK and BRAF	Dabrafenib + Trametinib	1	BRAF-K601E	1	At 2 months: 1 SD	0 (0)
Grousin et al. [68]	NTRK	Larotrectinib	1	EML4-NTRK3	1	At 2 months: 1 PR	1 (100)
Leboulleux et al. [50]	MEK and BRAF	Dabrafenib + Trametinib	21	BRAF-V600E	20	At 6 months: 8 PR, 11 SD, 1 PD	8 (38)
	MEK	Trametinib	10	RAS	6	At 6 months: 2 PR, 7 SD, 1 PD	2 (20)
Lee et al. [70]	NTRK	Larotrectinib	1	TPR-NTRK1	1	At 21 months: 1 CR	1 (100)
	RET	Selpercaptinib	1	CCDC6-RET	1	At 1 months: 1 PR	1 (100)
Grousin et al. [69]	RET	Selpercaptinib	1	NCOA4-RET	1	\	\
Bogsbud et al. [73]	BRAF	Dabrafenib	1	BRAF-V600E	1	\	\
Busaidy et al. [67]	MEK and BRAF	Dabrafenib + Trametinib	27	BRAF-V600E	13	At 6 months: 8 PR, 19 PD	8 (30)
	BRAF	Dabrafenib	26	BRAF-V600E (25) BRAF-K601E (1)	11	At 6 months: 9 PR, 17 PD	9 (35)
Weber et al. [74]	MEK and/or BRAF	Trametinib	20	WT (14)	5	At 12 months: 1 PR, 5 SD, 1 PD	1 (14)
		Dabrafenib + Trametinib		BRAF-V600E (6)	2		

*N*, patient numbers; WT, wild type; PR, partial response; SD, stable disease; CR, complete response; PD, progressive disease.

**Table 2:** Ongoing clinical trails for redifferentiation treatment.

Identifier	Started Year	Drug Targets	Agents	Patients (*N*)	Oncogenic Driver
NCT02152995	2014	MEK	Trametinib	34	BRAF-V600E or RAS
NCT02145143	2014	BRAF	Vemurafenib	12	BRAF-V600E or RAS
NCT02041260	2014	VEGFR	Cabozantinib	43	BRAF-V600E
NCT02456701	2015	BRAF and ErbB3	Vemurafenib + KTN3379	7	RET fusion
NCT03244956	2017	MEK and BRAF	Dabrafenib + Trametinib	40	BRAF-V600E
NCT03506048	2019	VEGFR\FGFR	Lenvatinib	4	RAS
NCT04554680	2020	MEK and BRAF	Dabrafenib + Trametinib	5	\
NCT04554680	2020	MEK and BRAF	Dabrafenib + Trametinib	5	\
NCT04462471	2020	BRAF and PI3K	Vemurafenib + Copanlisib	8	\
NCT04858867	2022	VEGFR\FGFR	Lenvatinib	12	BRAF-V600E or RAS
NCT06007924	2023	MEK and FAK	Avutometinib + Defactinib	30	BRAF-V600E
NCT05668962	2023	RET	Selpercatinib	30	\
NCT06475989	2024	BRAF	Cabozantinib/Dabrafenib + Trametinib	264	BRAF-V600E or RAS
NCT06458036	2024	RET	Selpercatinib	13	BRAF-V600E
NCT06440850	2024	BRAF	Vemurafenib + Cobimetinib	21	BRAF-V600E
NCT05783323	2024	NTRK	Larotrectinib	13	\

*N*, patient numbers;

## Data Availability

Not applicable.

## Abbreviations

AKT, Protein Kinase B; ATC, Anaplastic Thyroid Carcinoma; BRAF, B-Raf proto-oncogene; DNMT1, DNA (cytosine-5)-methyltransferase 1; DTC, Differentiated Thyroid Carcinoma; ERK, Extracellular Signal-Regulated Kinase; FTC, Follicular Thyroid Carcinoma; HER, Human Epidermal Growth Factor Receptor; MAPK, Mitogen-Activated Protein Kinase; MEK, Mitogen-activated Extracellular Signal-regulated Kinase; mTOR, Mammalian Target of Rapamycin; NOX4, NADPH Oxidase 4PDTC, Poorly Differentiated Thyroid Carcinoma; PAX8, Paired Box 8; PI3K, Phosphoinositide 3-Kinase; PKA, Protein Kinase A; PTEN, Phosphatase And Tensin Homolog; PTC, Papillary Thyroid Carcinoma; RAS, Rat SarcomaRET, Rearranged during Transfection; ROS, Reactive Oxygen Species; SMAD3, SMAD Family Member 3; TLR, Toll-like Receptors; Tg, Thyroglobulin; TSH, Thyroid-Stimulating Hormone.

## References

[1] Siegel R.L., Miller K.D., Wagle N.S., Jemal A. (2023). Cancer Statistics, 2023. CA Cancer J. Clin..

[2] Baloch Z.W., Asa S.L., Barletta J.A., Ghossein R.A., Juhlin C.C., Jung C.K., LiVolsi V.A., Papotti M.G., Sobrinho-Simões M., Tallini G. (2022). Overview of the 2022 WHO Classification of Thyroid Neoplasms. Endocr. Pathol..

[3] Fagin J.A. (1992). Genetic Basis of Endocrine Disease 3: Molecular Defects in Thyroid Gland Neoplasia. J. Clin. Endocrinol. Metab..

[4] Pozdeyev N., Gay L.M., Sokol E.S., Hartmaier R., Deaver K.E., Davis S., French J.D., Borre P.V., LaBarbera D.V., Tan A.-C. (2018). Genetic Analysis of 779 Advanced Differentiated and Anaplastic Thyroid Cancers. Clin. Cancer Res..

[5] Landa I., Pozdeyev N., Korch C., Marlow L., Smallridge R., Copland J., Henderson Y., Lai S., Clayman G., Onoda N. (2019). Comprehensive Genetic Characterization of Human Thyroid Cancer Cell Lines: A Validated Panel for Preclinical Studies. Clin. Cancer Res..

[6] Agrawal N., Akbani R., Aksoy B.A., Ally A., Arachchi H., Asa S., Auman J., Balasundaram M., Balu S., Baylin S. (2014). Integrated Genomic Characterization of Papillary Thyroid Carcinoma. Cell.

[7] Mitsutake N., Knauf J.A., Mitsutake S., Mesa C., Zhang L., Fagin J.A. (2005). Conditional BRAFV600E Expression Induces DNA Synthesis, Apoptosis, Dedifferentiation, and Chromosomal Instability in Thyroid PCCL3 Cells. Cancer Res..

[8] Santoro M., Moccia M., Federico G., Carlomagno F. (2020). RET Gene Fusions in Malignancies of the Thyroid and Other Tissues. Genes.

[9] Santoro M., Papotti M., Chiappetta G., Garcia-Rostan G., Volante M., Johnson C., Camp R.L., Pentimalli F., Monaco C., Herrero A. (2002). RET Activation and Clinicopathologic Features in Poorly Differentiated Thyroid Tumors. J. Clin. Endocrinol. Metab..

[10] Franco A.T., Ricarte-Filho J.C., Isaza A., Jones Z., Jain N., Mostoufi-Moab S., Surrey L., Laetsch T.W., Li M.M., DeHart J.C. (2022). Fusion Oncogenes Are Associated with Increased Metastatic Capacity and Persistent Disease in Pediatric Thyroid Cancers. J. Clin. Oncol..

[11] Morton L.M., Karyadi D.M., Stewart C., Bogdanova T.I., Dawson E.T., Steinberg M.K., Dai J., Hartley S.W., Schonfeld S.J., Sampson J.N. (2021). Radiation-Related Genomic Profile of Papillary Thyroid Carcinoma after the Chernobyl Accident. Science.

[12] Scholfield D.W., Fitzgerald C.W.R., Boe L.A., Eagan A., Levyn H., Xu B., Tuttle R.M., Fagin J.A., Shaha A.R., Shah J.P. (2024). Defining the Genomic Landscape of Diffuse Sclerosing Papillary Thyroid Carcinoma: Prognostic Implications of RET Fusions. Ann. Surg. Oncol..

[13] Nikiforov Y.E., Nikiforova M.N. (2011). Molecular Genetics and Diagnosis of Thyroid Cancer. Nat. Rev. Endocrinol..

[14] Landa I., Ibrahimpašić T., Boucai L., Sinha R., Knauf J., Shah R.H., Dogan S., Ricarte-Filho J., Krishnamoorthy G.P., Xu B. (2016). Genomic and Transcriptomic Hallmarks of Poorly Differentiated and Anaplastic Thyroid Cancers. J. Clin. Investig..

[15] Saqcena M., Leandro-Garcia L.J., Maag J.L.V., Tchekmedyian V., Krishnamoorthy G.P., Tamarapu P.P., Tiedje V., Reuter V., Knauf J.A., de Stanchina E. (2021). SWI/SNF Complex Mutations Promote Thyroid Tumor Progression and Insensitivity to Redifferentiation Therapies. Cancer Discov..

[16] Landa I., Cabanillas M.E. (2024). Genomic alterations in thyroid cancer: Biological and clinical insights. Nat. Rev. Endocrinol..

[17] Charles R.-P., Silva J., Iezza G., Phillips W.A., McMahon M. (2014). Activating BRAF and PIK3CA Mutations Cooperate to Promote Anaplastic Thyroid Carcinogenesis. Mol. Cancer Res. MCR.

[18] McFadden D., Vernon A., Santiago P.M., Martinez-McFaline R., Bhutkar A., Crowley D., McMahon M., Sadow P., Jacks T. (2014). P53 Constrains Progression to Anaplastic Thyroid Carcinoma in a Braf-Mutant Mouse Model of Papillary Thyroid Cancer. Proc. Natl. Acad. Sci. USA.

[19] Garcia-Rendueles M.E., Ricarte-Filho J.C., Untch B.R., Landa I., Knauf J.A., Voza F., Smith V.E., Ganly I., Taylor B.S., Persaud Y. (2015). NF2 Loss Promotes Oncogenic RAS-Induced Thyroid Cancers via YAP-Dependent Transactivation of RAS Proteins and Sensitizes Them to MEK Inhibition. Cancer Discov..

[20] Champa D., Russo M.A., Liao X.-H., Refetoff S., Ghossein R.A., Cristofano A.D. (2014). Obatoclax Overcomes Resistance to Cell Death in Aggressive Thyroid Carcinomas by Countering Bcl2a1 and Mcl1 Overexpression. Endocr. Relat. Cancer.

[21] Arciuch V.G.A., Russo M.A., Dima M., Kang K.S., Dasrath F., Liao X.-H., Refetoff S., Montagna C., Cristofano A.D. (2011). Thyrocyte-Specific Inactivation of P53 and Pten Results in Anaplastic Thyroid Carcinomas Faithfully Recapitulating Human Tumors. Oncotarget.

[22] Riesco-Eizaguirre G., Rodríguez I., De la Vieja A., Costamagna E., Carrasco N., Nistal M., Santisteban P. (2009). The BRAFV600E Oncogene Induces Transforming Growth Factor β Secretion Leading to Sodium Iodide Symporter Repression and Increased Malignancy in Thyroid Cancer. Cancer Res..

[23] Xing M. (2013). Molecular Pathogenesis and Mechanisms of Thyroid Cancer. Nat. Rev. Cancer.

[24] Mancikova V., Buj R., Castelblanco E., Inglada-Pérez L., Diez A., de Cubas A.A., Curras-Freixes M., Maravall F.X., Mauricio D., Matias-Guiu X. (2014). DNA Methylation Profiling of Well-Differentiated Thyroid Cancer Uncovers Markers of Recurrence Free Survival. Int. J. Cancer.

[25] Bisarro Dos Reis M., Barros-Filho M.C., Marchi F.A., Beltrami C.M., Kuasne H., Pinto C.A.L., Ambatipudi S., Herceg Z., Kowalski L.P., Rogatto S.R. (2017). Prognostic Classifier Based on Genome-Wide DNA Methylation Profiling in Well-Differentiated Thyroid Tumors. J. Clin. Endocrinol. Metab..

[26] Ravi N., Yang M., Mylona N., Wennerberg J., Paulsson K. (2020). Global RNA Expression and DNA Methylation Patterns in Primary Anaplastic Thyroid Cancer. Cancers.

[27] Puppin C., Passon N., Lavarone E., Di Loreto C., Frasca F., Vella V., Vigneri R., Damante G. (2011). Levels of Histone Acetylation in Thyroid Tumors. Biochem. Biophys. Res. Commun..

[28] Catalano M.G., Fortunati N., Pugliese M., Poli R., Bosco O., Mastrocola R., Aragno M., Boccuzzi G. (2006). Valproic Acid, a Histone Deacetylase Inhibitor, Enhances Sensitivity to Doxorubicin in Anaplastic Thyroid Cancer Cells. J. Endocrinol..

[29] Visone R., Russo L., Pallante P., De Martino I., Ferraro A., Leone V., Borbone E., Petrocca F., Alder H., Croce C.M. (2007). MicroRNAs (miR)-221 and miR-222, Both Overexpressed in Human Thyroid Papillary Carcinomas, Regulate p27Kip1 Protein Levels and Cell Cycle. Endocr. Relat. Cancer.

[30] Geraldo M.V., Yamashita A.S., Kimura E.T. (2012). MicroRNA miR-146b-5p Regulates Signal Transduction of TGF-β by Repressing SMAD4 in Thyroid Cancer. Oncogene.

[31] Riesco-Eizaguirre G., Wert-Lamas L., Perales-Patón J., Sastre-Perona A., Fernández L.P., Santisteban P. (2015). The miR-146b-3p/PAX8/NIS Regulatory Circuit Modulates the Differentiation Phenotype and Function of Thyroid Cells during Carcinogenesis. Cancer Res..

[32] Ramírez-Moya J., Wert-Lamas L., Santisteban P. (2018). MicroRNA-146b Promotes PI3K/AKT Pathway Hyperactivation and Thyroid Cancer Progression by Targeting PTEN. Oncogene.

[33] Li H., Yang H., Wen D., Luo Y., Liang C., Pan D., Ma W., Chen G., He Y., Chen J. (2017). Overexpression of LncRNA HOTAIR Is Associated with Poor Prognosis in Thyroid Carcinoma: A Study Based on TCGA and GEO Data. Horm. Metab. Res..

[34] Ma B., Xu W., Wei W., Wen D., Lu Z., Yang S., Chen T., Wang Y., Wang Y., Ji Q. (2018). Clinicopathological and Survival Outcomes of Well-Differentiated Thyroid Carcinoma Undergoing Dedifferentiation: A Retrospective Study from FUSCC. Int. J. Endocrinol..

[35] Kim H., Kwon H., Moon B.-I. (2022). Predictors of Recurrence in Patients with Papillary Thyroid Carcinoma: Does Male Sex Matter?. Cancers.

[36] Sung H., Ferlay J., Siegel R.L., Laversanne M., Soerjomataram I., Jemal A., Bray F. (2021). Global Cancer Statistics 2020: GLOBOCAN Estimates of Incidence and Mortality Worldwide for 36 Cancers in 185 Countries. CA Cancer J. Clin..

[37] Rahbari R., Zhang L., Kebebew E. (2010). Thyroid Cancer Gender Disparity. Future Oncol. Lond. Engl..

[38] Bible K., Kebebew E., Brierley J., Brito J., Cabanillas M., Clark T.J.E., Cristofano A.D., Foote R., Giordano T., Kasperbauer J. (2021). American Thyroid Association Guidelines for Management of Patients with Anaplastic Thyroid Cancer. Thyroid.

[39] Ito Y., Miyauchi A., Fujishima M., Noda T., Sano T., Sasaki T., Kishi T., Nakamura T. (2023). Thyroid-Stimulating Hormone, Age, and Tumor Size Are Risk Factors for Progression During Active Surveillance of Low-Risk Papillary Thyroid Microcarcinoma in Adults. World J. Surg..

[40] Kim D.H., Kim S.W., Basurrah M.A., Lee J., Hwang S.H. (2023). Diagnostic Performance of Six Ultrasound Risk Stratification Systems for Thyroid Nodules: A Systematic Review and Network Meta-Analysis. AJR Am. J. Roentgenol..

[41] Hahn S.Y., Shin J.H. (2016). Description and Comparison of the Sonographic Characteristics of Poorly Differentiated Thyroid Carcinoma and Anaplastic Thyroid Carcinoma. J. Ultrasound Med..

[42] Glazer D.I., Brown R.K.J., Wong K.K., Savas H., Gross M.D., Avram A.M. (2013). SPECT/CT Evaluation of Unusual Physiologic Radioiodine Biodistributions: Pearls and Pitfalls in Image Interpretation. RadioGraphics.

[43] Sakulpisuti C., Charoenphun P., Chamroonrat W. (2022). Positron Emission Tomography Radiopharmaceuticals in Differentiated Thyroid Cancer. Mol. Basel Switz..

[44] Durante C., Hegedüs L., Czarniecka A., Paschke R., Russ G., Schmitt F., Soares P., Solymosi T., Papini E. (2023). European Thyroid Association Clinical Practice Guidelines for thyroid nodule management. Eur. Thyroid J..

[45] Ricarte-Filho J., Ryder M., Chitale D., Rivera M., Heguy A., Ladanyi M., Janakiraman M., Solit D., Knauf J., Tuttle R. (2009). Mutational Profile of Advanced Primary and Metastatic Radioactive Iodine-Refractory Thyroid Cancers Reveals Distinct Pathogenetic Roles for BRAF, PIK3CA, and AKT1. Cancer Res..

[46] Xing M., Liu R., Liu X., Murugan A.K., Zhu G., Zeiger M.A., Pai S., Bishop J. (2014). BRAF V600E and TERT Promoter Mutations Cooperatively Identify the Most Aggressive Papillary Thyroid Cancer with Highest Recurrence. J. Clin. Oncol..

[47] Ringel M.D., Sosa J.A., Baloch Z., Bischoff L., Bloom G., Brent G.A., Brock P.L., Chou R., Flavell R.R., Goldner W. (2025). 2025 American Thyroid Association Management Guidelines for Adult Patients with Differentiated Thyroid Cancer. Thyroid.

[48] Morris L.G., Shaha A.R., Tuttle R.M., Sikora A.G., Ganly I. (2010). Tall-Cell Variant of Papillary Thyroid Carcinoma: A Matched-Pair Analysis of Survival. Thyroid.

[49] Ho A.L., Grewal R.K., Leboeuf R., Sherman E.J., Pfister D.G., Deandreis D., Pentlow K.S., Zanzonico P.B., Haque S., Gavane S. (2013). Selumetinib-Enhanced Radioiodine Uptake in Advanced Thyroid Cancer. N. Engl. J. Med..

[50] Leboulleux S., Benisvy D., Taieb D., Attard M., Bournaud C., Terroir-Cassou-Mounat M., Lacroix L., Anizan N., Schiazza A., Garcia M.E. (2023). MERAIODE: A Phase II Redifferentiation Trial with Trametinib and 131I in Metastatic Radioactive Iodine Refractory RAS Mutated Differentiated Thyroid Cancer. Thyroid.

[51] Leboulleux S., Dupuy C., Lacroix L., Attard M., Grimaldi S., Corre R., Ricard M., Nasr S., Berdelou A., Hadoux J. (2019). Redifferentiation of a *BRAF^K601E^*-Mutated Poorly Differentiated Thyroid Cancer Patient with Dabrafenib and Trametinib Treatment. Thyroid.

[52] Yoo S.-K., Song Y.S., Lee E., Hwang J., Kim H., Jung G., Kim Y.A., Kim S., Cho S., Won J. (2019). Integrative Analysis of Genomic and Transcriptomic Characteristics Associated with Progression of Aggressive Thyroid Cancer. Nat. Commun..

[53] Boucai L., Saqcena M., Kuo F., Grewal R.K., Socci N., Knauf J.A., Krishnamoorthy G.P., Ryder M., Ho A.L., Ghossein R.A. (2023). Genomic and Transcriptomic Characteristics of Metastatic Thyroid Cancers with Exceptional Responses to Radioactive Iodine Therapy. Clin. Cancer Res..

[54] Zhang Y., Xing Z., Liu T., Tang M., Mi L., Zhu J., Wu W., Wei T. (2022). Targeted therapy and drug resistance in thyroid cancer. Eur. J. Med. Chem..

[55] Chen A.Y., Jemal A., Ward E.M. (2009). Increasing Incidence of Differentiated Thyroid Cancer in the United States, 1988–2005. Cancer.

[56] Xing M., Haugen B.R., Schlumberger M. (2013). Progress in Molecular-Based Management of Differentiated Thyroid Cancer. Lancet.

[57] Nixon I.J., Whitcher M.M., Palmer F.L., Tuttle R.M., Shaha A.R., Shah J.P., Patel S.G., Ganly I. (2012). The Impact of Distant Metastases at Presentation on Prognosis in Patients with Differentiated Carcinoma of the Thyroid Gland. Thyroid.

[58] Durante C., Haddy N., Baudin E., Leboulleux S., Hartl D., Travagli J.P., Caillou B., Ricard M., Lumbroso J.D., De Vathaire F. (2006). Long-Term Outcome of 444 Patients with Distant Metastases from Papillary and Follicular Thyroid Carcinoma: Benefits and Limits of Radioiodine Therapy. J. Clin. Endocrinol. Metab..

[59] Ravera S., Reyna-Neyra A., Ferrandino G., Amzel L.M., Carrasco N. (2017). The Sodium/Iodide Symporter (NIS): Molecular Physiology and Preclinical and Clinical Applications. Annu. Rev. Physiol..

[60] Aashiq M., Silverman D.A., Na’ara S., Takahashi H., Amit M. (2019). Radioiodine-Refractory Thyroid Cancer: Molecular Basis of Redifferentiation Therapies, Management, and Novel Therapies. Cancers.

[61] Brown S.R., Hall A., Buckley H.L., Flanagan L., Gonzalez De Castro D., Farnell K., Moss L., Gregory R., Newbold K., Du Y. (2019). Investigating the Potential Clinical Benefit of Selumetinib in Resensitising Advanced Iodine Refractory Differentiated Thyroid Cancer to Radioiodine Therapy (SEL-I-METRY): Protocol for a Multicentre UK Single Arm Phase II Trial. BMC Cancer.

[62] Wadsley J., Ainsworth G., Coulson A.B., Garcez K., Moss L., Newbold K., Farnell K., Swain J., Howard H., Beasley M. (2023). Results of the SEL-I-METRY Phase II Trial on Resensitization of Advanced Iodine Refractory Differentiated Thyroid Cancer to Radioiodine Therapy. Thyroid.

[63] Dunn L.A., Sherman E.J., Baxi S.S., Tchekmedyian V., Grewal R.K., Larson S.M., Pentlow K.S., Haque S., Tuttle R.M., Sabra M.M. (2019). Vemurafenib Redifferentiation of BRAF Mutant, RAI-Refractory Thyroid Cancers. J. Clin. Endocrinol. Metab..

[64] Rothenberg S.M., McFadden D.G., Palmer E.L., Daniels G.H., Wirth L.J. (2015). Redifferentiation of iodine-refractory BRAF V600E-mutant metastatic papillary thyroid cancer with dabrafenib. Clin. Cancer Res..

[65] Subbiah V., Kreitman R., Wainberg Z., Cho J., Schellens J., Soria J., Wen P., Zielinski C., Cabanillas M., Urbanowitz G. (2018). Dabrafenib and Trametinib Treatment in Patients with Locally Advanced or Metastatic BRAF V600-Mutant Anaplastic Thyroid Cancer. J. Clin. Oncol..

[66] Jaber T., Waguespack S.G., Cabanillas M.E., Elbanan M., Vu T., Dadu R., Sherman S.I., Amit M., Santos E.B., Zafereo M. (2018). Targeted Therapy in Advanced Thyroid Cancer to Resensitize Tumors to Radioactive Iodine. J. Clin. Endocrinol. Metab..

[67] Busaidy N.L., Konda B., Wei L., Wirth L.J., Devine C., Daniels G.A., DeSouza J.A., Poi M., Seligson N.D., Cabanillas M.E. (2022). Dabrafenib Versus Dabrafenib + Trametinib in BRAF-Mutated Radioactive Iodine Refractory Differentiated Thyroid Cancer: Results of a Randomized, Phase 2, Open-Label Multicenter Trial. Thyroid.

[68] Groussin L., Clerc J., Huillard O. (2020). Larotrectinib-Enhanced Radioactive Iodine Uptake in Advanced Thyroid Cancer. N. Engl. J. Med..

[69] Groussin L., Bessiene L., Arrondeau J., Garinet S., Cochand-Priollet B., Lupo A., Zerbit J., Clerc J., Huillard O. (2021). Selpercatinib-Enhanced Radioiodine Uptake in RET-Rearranged Thyroid Cancer. Thyroid.

[70] Lee Y.A., Lee H., Im S.-W., Song Y.S., Oh D.-Y., Kang H.J., Won J.-K., Jung K.C., Kwon D., Chung E.-J. (2021). 2021 NTRK and RET Fusion-Directed Therapy in Pediatric Thyroid Cancer Yields a Tumor Response and Radioiodine Uptake. J. Clin. Investig..

[71] Huillard O., Tenenbaum F., Clerc J., Goldwasser F., Groussin L. (2017). Restoring Radioiodine Uptake in BRAF V600E-Mutated Papillary Thyroid Cancer. J. Endocr. Soc..

[72] Iravani A., Solomon B., Pattison D.A., Jackson P., Ravi Kumar A., Kong G., Hofman M.S., Akhurst T., Hicks R.J. (2019). Mitogen-Activated Protein Kinase Pathway Inhibition for Redifferentiation of Radioiodine Refractory Differentiated Thyroid Cancer: An Evolving Protocol. Thyroid.

[73] Bogsrud T., Jacobsen M., Durski J., Larsen E., Engelsen O., Haskjold O.I., Castillejo M., Bach-Gansmo T., Nostrand D.V. (2023). Letter to the Editor: Repeat Redifferentiation of Radioiodine Refractory BRAFV600E Mutated Thyroid Cancer with Dabrafenib. Thyroid.

[74] Weber M., Kersting D., Riemann B., Brandenburg T., Führer-Sakel D., Grünwald F., Kreissl M.C., Dralle H., Weber F., Schmid K.W. (2022). Enhancing Radioiodine Incorporation into Radioiodine-Refractory Thyroid Cancer with MAPK Inhibition (ERRITI): A Single-Center Prospective Two-Arm Study. Clin. Cancer Res..

[75] Montero-Conde C., Ruiz-Llorente S., Dominguez J.M., Knauf J.A., Viale A., Sherman E.J., Ryder M., Ghossein R.A., Rosen N., Fagin J.A. (2013). Relief of Feedback Inhibition of HER3 Transcription by RAF and MEK Inhibitors Attenuates Their Antitumor Effects in BRAF Mutant Thyroid Carcinomas. Cancer Discov..

[76] Tchekmedyian V., Dunn L., Sherman E., Baxi S.S., Grewal R.K., Larson S.M., Pentlow K.S., Haque S., Tuttle R.M., Sabra M.M. (2022). Enhancing Radioiodine Incorporation in BRAF-Mutant, Radioiodine-Refractory Thyroid Cancers with Vemurafenib and the Anti-ErbB3 Monoclonal Antibody CDX-3379: Results of a Pilot Clinical Trial. Thyroid.

[77] Plantinga T.S., Heinhuis B., Gerrits D., Netea M.G., Joosten L.A.B., Hermus A.R.M.M., Oyen W.J.G., Schweppe R.E., Haugen B.R., Boerman O.C. (2014). mTOR Inhibition Promotes TTF1-Dependent Redifferentiation and Restores Iodine Uptake in Thyroid Carcinoma Cell Lines. J. Clin. Endocrinol. Metab..

[78] Sherman E.J., Dunn L.A., Ho A.L., Baxi S.S., Ghossein R.A., Fury M.G., Haque S., Sima C.S., Cullen G., Fagin J.A. (2017). Phase 2 Study Evaluating the Combination of Sorafenib and Temsirolimus in the Treatment of Radioactive Iodine-Refractory Thyroid Cancer. Cancer.

[79] Hamidi S., Iyer P.C., Dadu R., Gule-Monroe M.K., Maniakas A., Zafereo M.E., Wang J.R., Busaidy N.L., Cabanillas M.E. (2024). Checkpoint Inhibition in Addition to Dabrafenib/Trametinib for BRAFV600E-Mutated Anaplastic Thyroid Carcinoma. Thyroid.

[80] Gui L., Zhu Y., Li X., He X., Ma T., Cai Y., Liu S. (2023). Case Report: Complete Response of an Anaplastic Thyroid Carcinoma Patient with NRAS Q61R/BRAF D594N Mutations to the Triplet of Dabrafenib, Trametinib and PD-1 Antibody. Front. Immunol..

[81] Tepmongkol S., Keelawat S., Honsawek S., Ruangvejvorachai P. (2008). Rosiglitazone Effect on Radioiodine Uptake in Thyroid Carcinoma Patients with High Thyroglobulin but Negative Total Body Scan: A Correlation with the Expression of Peroxisome Proliferator-Activated Receptor-Gamma. Thyroid.

[82] Simon D., Körber C., Krausch M., Segering J., Groth P., Görges R., Grünwald F., Müller-Gärtner H.W., Schmutzler C., Köhrle J. (2002). Clinical Impact of Retinoids in Redifferentiation Therapy of Advanced Thyroid Cancer: Final Results of a Pilot Study. Eur. J. Nucl. Med. Mol. Imaging.

[83] Dang H., Sui M., He Q., Xie J., Liu Y., Hou P., Ji M. (2023). Pin1 Inhibitor API-1 Sensitizes BRAF-Mutant Thyroid Cancers to BRAF Inhibitors by Attenuating HER3-Mediated Feedback Activation of MAPK/ERK and PI3K/AKT Pathways. Int. J. Biol. Macromol..

[84] Lasolle H., Schiavo A., Tourneur A., Gillotay P., de Faria da Fonseca B., Ceolin L., Monestier O., Aganahi B., Chomette L., Kizys M.M.L. (2024). 2024 Dual Targeting of MAPK and PI3K Pathways Unlocks Redifferentiation of Braf-Mutated Thyroid Cancer Organoids. Oncogene.

[85] Pita J.M., Raspé E., Coulonval K., Decaussin-Petrucci M., Tarabichi M., Dom G., Libert F., Craciun L., Wicquart L., Leteurtre E. (2023). CDK4 Phosphorylation Status and Rational Use for Combining CDK4/6 and BRAF/MEK Inhibition in Advanced Thyroid Carcinomas. Front. Endocrinol..

[86] Azouzi N., Cailloux J., Cazarin J.M., Knauf J.A., Cracchiolo J., Al Ghuzlan A., Hartl D., Polak M., Carré A., El Mzibri M. (2017). NADPH Oxidase NOX4 Is a Critical Mediator of BRAF^V600E^-Induced Downregulation of the Sodium/Iodide Symporter in Papillary Thyroid Carcinomas. Antioxid. Redox Signal..

[87] Liu Y.-Y., Zhang X., Ringel M.D., Jhiang S.M. (2012). Modulation of Sodium Iodide Symporter Expression and Function by LY294002, Akti-1/2 and Rapamycin in Thyroid Cells. Endocr. Relat. Cancer.

[88] Cheng W., Liu R., Zhu G., Wang H., Xing M. (2016). Robust Thyroid Gene Expression and Radioiodine Uptake Induced by Simultaneous Suppression of BRAF V600E and Histone Deacetylase in Thyroid Cancer Cells. J. Clin. Endocrinol. Metab..

[89] Choi Y.W., Kim H.-J., Kim Y.H., Park S.H., Chwae Y.J., Lee J., Soh E.Y., Kim J.-H. (2014). B-RafV600E Inhibits Sodium Iodide Symporter Expression via Regulation of DNA Methyltransferase 1. Exp. Mol. Med..

[90] Li L., Lv B., Chen B., Guan M., Sun Y., Li H., Zhang B., Ding C., He S., Zeng Q. (2015). Inhibition of miR-146b Expression Increases Radioiodine-Sensitivity in Poorly Differential Thyroid Carcinoma via Positively Regulating NIS Expression. Biochem. Biophys. Res. Commun..

[91] Wächter S., Wunderlich A., Greene B.H., Roth S., Elxnat M., Fellinger S.A., Verburg F.A., Luster M., Bartsch D.K., Di Fazio P. (2018). Selumetinib Activity in Thyroid Cancer Cells: Modulation of Sodium Iodide Symporter and Associated miRNAs. Int. J. Mol. Sci..

[92] Oh J.M., Rajendran R.L., Gangadaran P., Hong C.M., Jeong J.H., Lee J., Ahn B.-C. (2022). Targeting GLI1 Transcription Factor for Restoring Iodine Avidity with Redifferentiation in Radioactive-Iodine Refractory Thyroid Cancers. Cancers.

[93] Eisenhauer E.A., Therasse P., Bogaerts J., Schwartz L.H., Sargent D., Ford R., Dancey J., Arbuck S., Gwyther S., Mooney M. (2009). New Response Evaluation Criteria in Solid Tumours: Revised RECIST Guideline (Version 1.1). Eur. J. Cancer.

[94] Wahl R.L., Jacene H., Kasamon Y., Lodge M.A. (2009). From RECIST to PERCIST: Evolving Considerations for PET Response Criteria in Solid Tumors. J. Nucl. Med..

[95] Balakirouchenane D., Seban R., Groussin L., Puszkiel A., Cottereau A.S., Clerc J., Vidal M., Goldwasser F., Arrondeau J., Blanchet B. (2023). Pharmacokinetics/Pharmacodynamics of Dabrafenib and Trametinib for Redifferentiation and Treatment of Radioactive Iodine-Resistant Mutated Advanced Differentiated Thyroid Cancer. Thyroid.

